# Withdrawal of antihypertensive therapy in people with dementia: feasibility study

**DOI:** 10.1186/s40814-017-0221-0

**Published:** 2018-01-09

**Authors:** Veronika van der Wardt, Jennifer K. Burton, Simon Conroy, Tomas Welsh, Pip Logan, Jaspal Taggar, Lukasz Tanajewski, John Gladman

**Affiliations:** 1 Division of Rehabilitation and Ageing, School of Medicine, University of Nottingham, Nottingham, NG7 2UH UK; 2 Centre for Cognitive Ageing and Cognitive Epidemiology & The Alzheimer Scotland Dementia Research Centre, Royal Infirmary of Edinburgh, Edinburgh, EH16 4SA UK; 3 Department of Health Sciences, University of Leicester, Leicester, LE1 6TP UK; 4 Research Institute for the Care of Older People, Combe Parke, Bath, BA1 3NG UK; 5 Division of Primary Care School of Medicine, University of Nottingham, Nottingham, NG7 2UH UK; 6 Department of Economics, Kozminski University, Warsaw, Poland; 7 Division of Pharmacy Practice and Policy, School of Pharmacy, University of Nottingham, Nottingham, NG7 2RD UK

**Keywords:** Hypertension, Dementia, Antihypertensive medication, Withdrawal, Cessation, Feasibility study, Patient experience, Recruitment, Primary care

## Abstract

**Background:**

This study explored the feasibility of a randomised controlled withdrawal trial of antihypertensive medication in normotensive people with dementia. Feasibility aspects included response, recruitment, exclusion and drop-out rates, suitability of outcome measures, acceptability of study procedures and an indicative economic evaluation for a randomised controlled trial.

**Methods:**

A cohort study attempting the withdrawal of antihypertensive drugs where appropriate and a feasibility study of home-based blood pressure monitoring, in people with dementia treated for hypertension, was undertaken. Interviews with participants and carers and an indicative economic evaluation were also undertaken.

**Results:**

Three hundred and sixty-two primary care practices in the East Midlands were contacted of which only 41 (11% (95%CI 8–15%)) agreed to support the study. These 41 practices posted 940 letters to potential participants. Thirty participants were enrolled in the cohort study of whom 9 were eligible for the antihypertensive withdrawal programme, 20 participated in a home blood pressure monitoring sub-group analysis and 12 took part in an interview study. Twenty-two of those enrolled in the cohort study were followed up at 6 months. The withdrawal programme was acceptable to participants and general practitioners (GPs). The study procedures including assessments and home blood pressure monitoring were acceptable to the participants and their carers. The economic evaluation was not possible.

**Conclusion:**

A withdrawal trial of antihypertensive medication in normotensive people with dementia may not be feasible in the UK because of low recruitment rates.

## Background

People with dementia are at greater risk of the adverse effects of antihypertensive medications, such as falls and syncope, than those without dementia due to their increased frailty and autonomic instability [1]. Antihypertensive medication can contribute to polypharmacy, which is particularly common and troublesome in people with dementia partly due to the cumulative burden of anticholinergic activity [2]. These increased concerns about the use of antihypertensive therapy in people with dementia are of particular importance because the balance of benefits and harms from antihypertensive therapy is unclear in this population [3]. Given that it is reported that antihypertensive medication can be discontinued for 1 year or more without hypertension returning in 44–66% of people with well-controlled hypertension [4–6], a large randomised controlled trial (RCT) of antihypertensive withdrawal to examine the balance of benefits and harms in this population may be justified.

We aimed to conduct a study to investigate the feasibility of undertaking a larger randomised trial of the withdrawal of antihypertensive medication in patients with dementia. The research questions of this study were as follows:

1. Can suitable and adequate numbers of potential participants for a RCT be identified?
2. Will suitable and adequate numbers of potential participants give consent to a RCT?
3. Are the trial procedures for a RCT including communication, consent procedure, documentation and assessments acceptable to participants?
4. Are the proposed trial baseline and outcomes measures suitable (i.e. minimal missing data)?
5. Can the intervention to withdraw antihypertensive medication be put into practice adequately for testing in a RCT and in anticipation of subsequent wider adoption?
6. What is the evidence in the scientific literature from a health economics perspective that supports or limits the justifiability of a RCT?

## Methods

### Research ethics

The study was approved by the West Midlands Health Research Ethics Committee [reference: 13/WM/0468]. Participants provided written informed consent or consultees provided written informed agreement.

### Methodology

To answer these six research questions, our study comprised the following elements: a cohort study comprising medication withdrawal, home blood pressure monitoring and an interview sub-study was undertaken. The medication withdrawal sub-study included plans for an indicative economic evaluation.

The cohort study was conducted using identification and recruitment processes from primary care databases that would be used in a subsequent RCT. We aimed to study all the potential participants identified from general practitioner (GP) databases who would meet the selection criteria for a subsequent RCT. Identification of potential participants via memory clinics was explored but not pursued because the local memory clinics were at research capacity and would not necessarily engage GPs who would be essential to oversee the eventual withdrawal of antihypertensive medication. Identification of potential participants via a national website specifically to support the engagement of patients in research (Join Dementia Research website [7]) was explored but led only to one potential participant who expressed interest but who did not consent to participate.

As we suspected that recruitment via GP databases was likely to identify some people who were ineligible for withdrawal due to inadequate recording in those databases of the selection criteria, we undertook a further assessment of those enrolled in the cohort study to assess their true eligibility for withdrawal of their antihypertensive medications, whilst evaluating the feasibility of the follow-up RCT trial procedures in the overall cohort to answer research questions one, two and four. An antihypertensive therapy withdrawal procedure, developed for this study, was applied to those who met the selection criteria after further clinical assessment (the withdrawal sub-study) to answer research question five.

The feasibility of home-based blood pressure monitoring (HBPM) in a sub-group of cohort study participants was tested to contribute to research question five, as HBPM is part of best clinical practice [8] and was suggested to be incorporated in hypertension trials [9], and we anticipated that it could ensure greater safety during antihypertensive therapy withdrawal than weekly or monthly clinic measurements.

We undertook an interview study with a sub-group of the participants to answer research question three and explore cohort study participants’ views and experience of research participation.

We attempted to conduct an indicative economic evaluation of the withdrawal procedure, combining the results of a literature analysis and the results of the cohort study in a Markov model, to answer research question six.

### Cohort study: identification of potential participants

Potential participants were identified through GP practices in Nottinghamshire, Leicestershire and Derbyshire (East Midlands, UK). GP practices, contacted by phone and/or email, were provided with study information and asked to assist in the recruitment process. Practices that did so were reimbursed £70 independent of subsequent recruitment. Practices were approached via the Clinical Research Network (CRN) and via the research team directly when the CRN did not have contacts to the practice. Practice responses and reasons for not assisting the recruitment process were recorded.

General practices that agreed to assist in the identification of potential participants were requested to screen their practice lists and databases for the following:

- Those with a diagnosis of dementia *and* a diagnosis of hypertension who were receiving antihypertensive treatment
- Who were aged 18 years or older
- Had no transient ischemic attack, stroke or myocardial infarction in the last 12 months
- Had no previous malignant hypertension, left ventricular failure or congestive cardiac failure
- Were not under-treated for hypertension (sitting blood pressure (BP), re-assessed by the research nurse at baseline by measuring the participant’s BP): systolic BP ≥ 150 mmHg or diastolic BP ≥ 90 mmHg on two separate occasion in people 80 years or over, or BP > 140/80 mmHg in people under 80 years of age, or BP > 130/80 mmHg in people with diabetes and chronic kidney disease (any state) or BP > 130/80 mmHg in people with chronic kidney disease (any stage) and proteinuria (ACR ≥ 70)

People identified from the screening of GP databases who met the above criteria were contacted by their practice by post, given information about the study and invited to contact the research team by post, email or telephone if they were interested in participating.

### Cohort study: recruitment of participants

Potential participants who expressed interest in the study were contacted by telephone to discuss the study and ensure that they were living with a carer. If interested in participating in the study, the research team sent further study information by post and arranged the baseline visit. The participant information sheet explained the purpose of the study, the potential frequency and duration of visits, and the procedures as well as potential risks and benefits of taking part. Carers were invited to participate in the interview study separately. Signed participant consent or consultee agreement was completed prior to the baseline assessment either from the participant with the mental capacity to do so (consent) or from the carer in cases where the participant did not have mental capacity (agreement). Participation in home blood pressure monitoring (HBPM) was optional and included in the consent/agreement form.

### Baseline and follow-up assessments

Follow-up took place 6 months after the baseline assessment irrespective of the lengths of withdrawal procedure. All recruited participants (apart from those who were excluded when found not to be on antihypertensive medication) were invited to complete follow-up assessments. Participants’ demographic characteristics were assessed at baseline only, and the National Health Service (NHS) use and potential side effects associated with antihypertensive therapy were recorded at both assessments. Based on a systematic scoping review of domains potentially affected by antihypertensive therapy [3], scales for cognition, depression, agitation, sleep quality and functional ability were selected. A quality of life scale was added to support the health economics evaluation.

The following assessment scales were used at baseline and follow-up assessment:

- Global cognition (Montreal Cognitive Assessment (MoCA); [10])
- Depression (Cornell scale; [11])
- Agitation (CMAI; [12])
- Sleep disturbances (NPI-SD; [13])
- Functional ability (Disability Assessment for Dementia (DAD); [14])
- Activities of daily living (Barthel index; [15])
- Quality of life (DEMQOL; [16])

Wherever possible, outcomes were ascertained directly from participants, but where cognition precluded outcome completion, proxy questionnaire versions were used. Answers reflecting the situation most accurately in the view of the research nurse were used to complete the questionnaires.

At baseline and follow-up assessments, blood pressure (BP) was measured twice on each arm after sitting for 5 min and standing. BP was measured on the arm with the lower sitting BP after lying down for 3 min and standing up for 1 min. Symptoms of orthostatic hypotension were recorded. A blood sample was taken at baseline to assess kidney function.

### Withdrawal programme

A research nurse confirmed that the inclusion and exclusion criteria used in the identification process were valid, applied a final section process to exclude those with over-treated and uncontrolled hypertension and collected the medical history and prescription data necessary to make the decision to attempt antihypertensive withdrawal.

A senior geriatrician from the research team used the results of these assessments and a summary of the participant’s medical history obtained from the GP to determine whether they were suitable for withdrawal of their antihypertensive medications and, if they were, drew up an individualised antihypertensive withdrawal plan. The antihypertensive withdrawal plan was developed by the study team [17] based upon guidance from previous studies, national prescribing guidance and NICE guidelines for the use of antihypertensive therapy. A key principle of the withdrawal plan was that BP medication should be withdrawn gradually with the duration of the process depending on the number and type of medication taken [8]. During the withdrawal procedure, each week, the nurse monitored the participants’ blood pressures, adverse events, symptoms related to antihypertensive medication use, change in medication and visits to or from health professionals. If antihypertensives were withdrawn, monitoring visits took place monthly. After every visit, the participant’s GP was informed of the results.

Participants’ GPs were informed of the withdrawal plans and were asked to agree to them. The research nurse undertook the implementation of the plan by taking and recording blood pressure measurements and informing the GP of what dose adjustments to antihypertensive therapy were required in line with the individualised plan. The ultimate decision to implement the withdrawal programme or to re-start antihypertensive therapy remained with the GP: the withdrawal procedure plans were advisory.

### Home blood pressure monitoring study

At recruitment to the cohort study, participants were asked if they would like to take part in a 1-week home blood pressure monitoring sub-study. Each participant who agreed to participate in the home blood pressure monitoring sub-study received a British Hypertension Society-approved Kinetik monitor with the appropriate cuff, a large letter version of its instruction booklet and a record sheet to note down all measurements. Following instructions on how to use the blood pressure monitor, the research nurse completed the first measurement and recorded the results. Following NICE guidelines for hypertension [8], participants were asked to complete the measurements for the rest of the week in the morning and in the evening, each time on both arms, either on their own or with the help of their relative.

### Interview study

Participants and their spouses in the cohort study were also invited to take part in an interview sub-study. Consent for interviews was sought from participants and carers separately. Recruitment to the interview study occurred until the analysis of the transcripts indicated that no new themes emerged. Semi-structured interviews were conducted by a research team member (VvdW) at each participant’s home. An interview schedule guided the interviews and outlined questions regarding the decision to take part and consent to the study, thoughts and feelings around stopping antihypertensive therapy and about the study procedures including home blood pressure monitoring. Participants and family members could choose if they wanted to complete the interviews separately or together.

### Indicative economic evaluation

A Markov model was planned to estimate the long-term health and economic effects of the withdrawal of antihypertensive therapy in people with dementia. The parameters required for such a model included the costs and outcomes (vascular event, progression of dementia, falls, etc) of usual care and care when withdrawal was undertaken and the likelihood of successful withdrawal. These parameters were to be drawn from previously published literature and the findings of the cohort study.

### Analyses

Descriptive statistics of the findings of the identification and recruitment processes were used to answer research questions 1 and 2 (identification and recruitment).

Research question 3 (acceptability) was answered from the interview study using qualitative analysis. Audio recordings of the interviews were transcribed by a professional transcriber into a word document, and data were imported into QSR Nvivo 11 software. The interviewer (VvdW) analysed the text for emerging themes, which served as guiding framework for coding and analysis. The analysis was a continuing process throughout the interview study. Quotes representing the themes were identified and compared with the transcript to be used in the appropriate context.

Research question 4 (suitability of measures) was answered by examining the completion rates at baseline. Mean systolic and diastolic BP levels for sitting measurements were calculated and described. Data for NHS service use, potential side effects of antihypertensive treatment (AHT) and kidney function were collected to explore practicability and acceptability of data collection but are not reported in the “Results” section. All quantitative analyses were completed in Stata version 13.

Research question 5 (practicality of intervention) was examined based on number of people eligible for the withdrawal procedure, descriptive statistics of these participants, time until hypertension returned and adverse events. The suitability of home-based blood pressure monitoring was investigated based on the number of participants completing this sub-study and the proportion of participants missing six or more measurements out of a total of 28 over 1 week.

There were no pre-planned analyses for research question 6.

## Results

### Identification and recruitment

Recruitment was completed between June 2014 and June 2015. Forty-one of 362 (11%) primary care practices in the East Midlands who were contacted agreed to support the study and screened their databases. Those who decided not to support the study and provided reasons (*n* = 112) indicated the following reasons:

- Too busy (*n* = 58, 52%)
- Undergoing practice or staff changes or were short staffed (*n* = 20, 17%)
- Not receiving sufficient funding for supporting the study (*n* = 10, 9%)
- Already supporting studies and at research capacity (*n* = 9, 8%)
- Not an appropriate practice for the study due to their patient list (*n* = 9, 8%)
- Not supporting research in general (*n* = 4, 4%)
- Not interested in supporting this particular study (*n* = 2, 2%)

The 41 supporting practices sent out a total of 940 letters (mean 23 per practice) from which a total of 68 replies (mean 1.7 per practice) were received from interested participants. Following the pre-interview telephone conversation, 30 out of 68 remained interested and met the eligibility criteria and agreed to an appointment for a baseline assessment. The remaining 38 did not want to participate (*n* = 22) or were not eligible (*n* = 16).

**Fig. 1:**
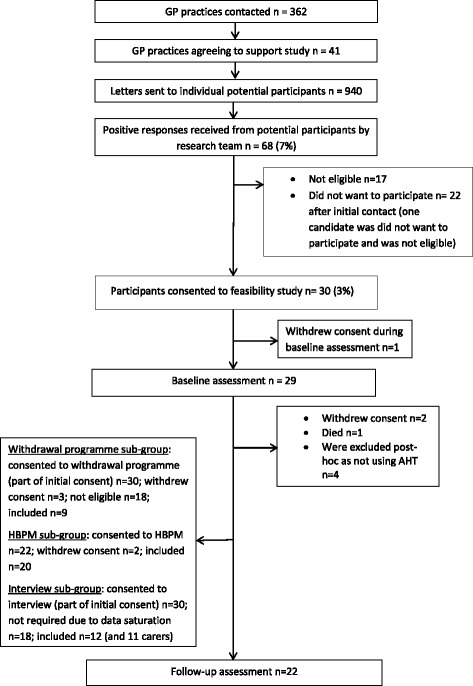
Participant flow

We recruited 30 participants diagnosed with dementia and hypertension (mean age 70.2 years (SD 6.9); range 56–93 years; women *n* = 13; mean years of education 12 (SD 4.3); ethnicity 93% white) and assessed 22 participants at follow-up. Losses occurred due to withdrawing consent or not meeting eligibility criteria (not using AHT). Three participants withdrew their consent, and one participant died after the baseline assessment. Four participants were not using AHT at the point of the baseline assessment. Figure 1 shows the participant flow.

### Withdrawal sub-group analysis

Nine participants (mean age 80.1 years (SD 5.2); five women; mean number of antihypertensive agents 1.6) were suitable for the withdrawal procedure. The mean baseline systolic BP of this group was 131 mmHg (SD 6.8), and the mean baseline diastolic BP was 72 mmHg (SD 10.6). There was no significant difference in the numbers of antihypertensive agents taken between those eligible for withdrawal and those not eligible.

None of the nine participants who completed the withdrawal procedure were both off antihypertensive therapy and normotensive at 6 months. Three participants whose blood pressures had reached the threshold for re-starting antihypertensive therapy were not taking them at follow-up, following the decision of their treating GP not to re-start them. The time between starting withdrawal and when blood pressure limits were exceeded ranged from 1 to 9 weeks (mean 2.4 weeks; SD 2.8). No adverse events related to the withdrawal procedure occurred.

### Home blood pressure sub-group analysis

In total, 20 participants completed the home blood pressure monitoring study. Four participants (20%) missed six or more measurements out of the total 28 measurements over the week.

Home blood pressure monitoring was new to the majority of participants but once they understood the instructions, they seemed to cope with it very well. One participant had minor difficulties completing the record sheet.

One participant and their relative used their own BP monitor, an Omron M3 device. Two participants experienced pain when using the home blood pressure monitor and withdrew from this part of the study.

### Acceptability of trial procedures

The quotes to support the following themes are presented in Table 1.

**Table 1:** Supporting quotes for acceptability of trial procedures

Participant/relative	Theme/sub-theme	Quote
Relative C	1—Participation and consent/ altruistic reasons	‘I’m all for any research … won’t benefit me but it’ll probably benefit somebody else.’
Participant D	2—Participation and consent/ altruistic reasons	‘I feel that I need help from you, so I want to give something back.’
Relative E	3—Participation and consent/ participation might reduce number of drugs	‘…anything to do with reducing the tablets is good.’
Participant F	3—Participation and consent/ participation might reduce number of drugs	‘I was hoping that they could reduce it [the medication].’
Participant B	3—Participation and consent/ participation might reduce number of drugs	‘…if you can go without any tablets, it surely has got to be better for you.’
Relative A	4—Participation and consent/ personal learning	‘…perhaps I’m learning things that I don’t know anything about.’
Relative E	5—Participation and consent/ worries but also hope	‘I was a bit dubious because I didn’t know which way it was going to go,… But when [the GP] confirmed it, he thought it’d be good, and I can, I was all right then.’
Relative G	5—Participation and consent/ worries but also hope	‘…if it can help him to be, you know, all right longer, it’s worth it’.
Relative H	6—Reflections of assessments and other study procedures /feeling comfortable	‘They [the questions] were alright…didn’t worry me at all.’
Participant I	6—Reflections of assessments and other study procedures/high number of questions	: ‘…a lot of them, a lot of questions…’
Relative J	6—Reflections of assessments and other study procedures/high number of questions	‘No, it’s fine, I mean, don’t mind us answering questions at all, you know, it don’t, you know, if it helps, it doesn’t matter, does it really. ’
Relative H	6—Reflections of assessments and other study procedures/high number of questions	‘No problem at all.’
Relative G	6—Reflections of assessments and other study procedures/ positive to have someone coming	‘It’s nice to know somebody’s there, that’s what…isn’t it? …somebody coming.. .’
Relative H	6—Reflections of assessments and other study procedures/ positive to have someone coming	‘I loved them coming’.
Relative K	6—Reflections of assessments and other study procedures/ memory questions	‘…some of the questions were referenced towards the memory problems… and [name of spouse] got a bit stressed out.’

#### Participation and consent (themes 1 and 2)

Several sub-themes for reasons to support the study and thoughts around consent emerged in the interviews: altruism, the possibility to reduce medication and personal learning. The expression of altruistic reasons was common with respondents acknowledging that the results of the feasibility study might not help themselves but might help others or research in general. One person also expressed the wish to give something back in return for the help that they had received.

Another sub-theme (theme 3) was the possibility that participation might reduce the number of drugs the participant had to take. This was perceived as a potentially positive consequence of the study. One participant also expressed a worry about taking tablets that might not be needed. Furthermore, personal learning (theme 4) and interest in the topic were mentioned as reasons to support the study. Some carers also expressed worries but also hope when discussion the initial thoughts about the study and their thoughts and feelings regarding consenting to the study (theme 5).

#### Assessments and other study procedures (theme 6)

In general, participants felt comfortable with the assessments. Some participants noticed that the baseline and follow-up assessments included a large number of questions, but did not comment negatively upon that. Some relatives also indicated that it was positive to have someone coming to their home. One participant did not like all the questions and the spouse indicated that the memory questions in particular made the participant uncomfortable.

### Suitability of measures

At baseline, 27 out of 30 participants (90%) completed all measurements. One participant completed all measures except the CMAI and two participants did not complete the questionnaires (one due to withdrawing consent, one completed only the demographic and health questionnaires). At follow-up, 20 out of 22 participants (91%) completed all measurements. For two participants, BP monitoring was too painful and two participants did not complete the MoCA.

Mean BP levels and scores of the questionnaires at baseline are shown in Table 2 in order to describe the cohort sample. The average duration of the baseline assessments was 153 min.

**Table 2:** Baseline measurements

	Baseline	
	*N*	
Mean systolic BP in mmHg (SD)	28*	141.5 (20.5)
Mean diastolic BP in mmHg (SD)	28*	74.9 (13.9)
MoCA mean score (SD)	28*	14.8 (6.3)
Barthel index mean (SD)	28*	82.5 (24.4)
DAD mean score (SD)	28*	57.8 (31.5)
Cornell mean score (SD)	28*	6.6 (5.8)
DemQoL mean score (SD)	28*	88.2 (16.3)
CMAI mean score (SD)	27*+	17.6 (5.9)
NPI-SD mean score (SD)	28*	1 (1.9)

*One participant only completed demographic and health questionnaires; +One participant did not complete CMAI

### Indicative economic evaluation

Because of the low number of participants, no useable data from the cohort study were obtained for economic evaluation parameters. Regarding the Markov model, the team determined that there was conflicting evidence about whether it is not known if antihypertensive therapies could have protective or harmful effects regarding cognition [18–21] and other events such as falls [1, 22, 23] and on how to design the detailed model structure; the data for such a model were not available from the literature. It was concluded that it was not possible to develop a Markov model on existing evidence.

## Discussion

Despite inviting 362 primary care practices in the East Midlands (approximate population 2.5 million) and sending 940 letters to potential participants, only nine participants were recruited for the withdrawal procedure. The trial procedures and assessments, including home blood pressure monitoring, were generally feasible and acceptable to participants. There was insufficient information to develop a useful economic model of the potential effects of withdrawing antihypertensive therapy.

There were two reasons for the low recruitment rate: the low rate of engagement of general practices willing to support the study (11%) and the low rate of recruitment of those who were identified (< 1% were suitable for withdrawal). The involvement of general practitioners GPs and hence recruitment via general practices was considered to be essential for the withdrawal study as GPs have responsibility for the management of antihypertensive therapy. The low engagement rate of general practices was seen despite the support from the Clinical Research Network, which is intended to support the conduct of research in the UK’s health service, and is likely to reflect the pressure which UK primary care practices experienced during the study period. Only a small number of practices indicated they did not want to support this study in particular (2%), but an additional 10% indicated that they would not receive enough compensation funds for this study. However, the feedback suggested that the lack of time and staff could affect research in GP practices in general. We have no reason to think that the engagement of general practices would be better in any other part of the UK although specific characteristics of the GP practices involved in the study were not investigated. A randomised controlled withdrawal trial conducted in a similar period in the Netherlands successfully recruited 385 participants from primary care practices within 26 months (DANTE [24]), although it had less stringent entry criteria than we applied. Thus, higher levels of general practice engagement and hence potential recruitment of participants might be possible in other countries or in the UK if conditions changed in the future. However, as < 1% of those contacted by their GPs in the current study were enrolled in the withdrawal programme, this low recruitment rate would still limit the feasibility of a RCT using these methods. Even taking all participants who consented into account, the recruitment rate in this study (3.2%) is considerably lower than the 11% of people with dementia that have been suggested would take part in drug trials [25]. A narrative review of barriers to prescribing and deprescribing in people with dementia identified fear of negative consequences of stopping medication, inability to change medication taking habits and established beliefs in benefits and harms of medication use [26]. These factors might have played a role in our study. Future studies may need a more personalised approach to encourage patients and their carers to participate, and this would require considerably more resources than the postal/telephone process used in this study.

The proposed RCT intervention—withdrawal of antihypertensive drugs—was acceptable to GPs, patients and their carers. The trial procedures were also acceptable, including the use of home blood pressure monitoring. Thus, if higher rates of general practice engagement and recruitment of participants were to be possible, then a trial of withdrawal of antihypertensive medications using these methods would be feasible. However, although this feasibility study was not designed and powered to estimate the proportion of participants who could withdraw antihypertensive and remain normotensive for a sustained period, the fact that none of the nine in whom withdrawal was attempted did so raises concerns that the methods we used selected a group of patients who would be unlikely to benefit and hence that a RCT using these methods would be unwarranted. The methods we used took into account the need in this vulnerable group of people to ensure their safety and was conducted in the context that guidelines for the treatment of hypertension [8] imply that those with dementia should be treated similarly to those without dementia and where these guidelines do not advise routinely attempting the withdrawal of antihypertensive drugs in patients who are normotensive. We also excluded those with end organ damage or recent vascular events that might put them at particular risk from recurrent hypertension. The withdrawal plan [17] was based on previous studies and hypertension guidelines for people without dementia as there was insufficient evidence to conclude that withdrawal should be different for people with dementia [27]. Furthermore, modern management of hypertension in primary care in the UK is incentivised to optimise BP control. For all these reasons, we are likely to have selected a group of stable patients with a low probability of being able to withdraw antihypertensive drugs successfully. Further studies of withdrawal of antihypertensive drugs in people with dementia might be more worthwhile if focussed upon those at particular risk such as those with severe dementia, in care homes, with low BPs on more than one antihypertensive agent.

## Conclusion

Given the potential difficulties highlighted in this study in the conduct of a RCT in this group of patients, an alternative approach to investigate the benefits and harms of antihypertensive drugs in people with dementia might be to examine large primary care datasets to ascertain the benefits and harms of antihypertensive treatment in people with dementia.

In summary, the low recruitment rates found in this feasibility imply that a large RCT using a similar method in the UK would not be feasible.

## Abbreviations

AHT: Antihypertensive treatment
BP: Blood pressure
GP: General practitioner
HBPM: Home blood pressure monitoring
MoCA: Montreal Cognitive Assessment
NHS: National Health Service
RCT: Randomised controlled trial
SD: Standard deviation
UK: United Kingdom

## References

[1] Eriksson S, Gustafson Y, Lundin-Olsson L (2008). Risk factors for falls in people with and without a diagnose of dementia living in residential care facilities: a prospective study. Arch Gerontol Geriatr.

[2] Davies EA, O’Mahony MS (2015). Adverse drug reactions in special populations—the elderly. Br J Clin Pharmacol.

[3] van der Wardt V, Logan P, Conroy S, Harwood R, Gladman J (2014). Antihypertensive treatment in people with dementia. J Am Med Dir Assoc.

[4] Beltman FW, Heesen WF, Kok RH, Smit AJ, May JF, de Graeff PA (1996). Predictive value of ambulatory blood pressure shortly after withdrawal of antihypertensive drugs in primary care patients. BMJ.

[5] Fernandez PG, Kim BK, Galway AB, Sharma JN (1983). Separation of essential hypertensive patients based on blood pressure responses after the withdrawal of antihypertensive agents by step-wise discriminant analysis. Curr Med Res Opin.

[6] Council MR (1986). Course of blood pressure in mild hypertensives after withdrawal of long term antihypertensive treatment. Medical Research Council working party on mild hypertension. Br Med J (Clin Res Ed).

[7] Research NIfH. Join Dementia Research: NIHR; 2016. Available from: https://www.joindementiaresearch.nihr.ac.uk/. Accessed 27 Dec 2017.

[8] National Clinical Guideline C (2011). National Institute for health and clinical excellence: guidance.

[9] Breaux-Shropshire TL, Judd E, Vucovich LA, Shropshire TS, Singh S (2015). Does home blood pressure monitoring improve patient outcomes? A systematic review comparing home and ambulatory blood pressure monitoring on blood pressure control and patient outcomes. Integr Blood Press Control.

[10] Larner AJ (2012). Screening utility of the Montreal Cognitive Assessment (MoCA): in place of—or as well as—the MMSE?. Int Psychogeriatr.

[11] Alexopoulos GS, Abrams RC, Young RC, Shamoian CA (1988). Cornell Scale for depression in dementia. Biol Psychiatry.

[12] Cohen-Mansfield J (1986). Agitated behaviors in the elderly II. Preliminary results in the cognitively deteriorated. J Am Geriatr Soc.

[13] Cummings JL, Mega M, Gray K, Rosenberg-Thompson S, Carusi DA, Gornbein J (1994). The neuropsychiatric inventory: comprehensive assessment of psychopathology in dementia. Neurology.

[14] Gelinas I, Gauthier L, McIntyre M, Gauthier S (1999). Development of a functional measure for persons with Alzheimer’s disease: the disability assessment for dementia. Am J Occup Ther.

[15] Mahoney FI, Barthel DW (1965). Functional evaluation: the Barthel index. Md State Med J.

[16] Smith SC, Lamping DL, Banerjee S, Harwood R, Foley B, Smith P (2005). Measurement of health-related quality of life for people with dementia: development of a new instrument (DEMQOL) and an evaluation of current methodology. Health Technol Assess.

[17] Harrison JK, Conroy S, Welsh T, Van der Wardt V, Gladman JRF (2016). Proposed antihypertensive medication withdrawal protocol.

[18] Forette F, Seux ML, Staessen JA, Thijs L, Babarskiene MR, Babeanu S (2002). The prevention of dementia with antihypertensive treatment: new evidence from the Systolic Hypertension in Europe (Syst-Eur) study. Arch Intern Med.

[19] in’t Veld BA, Ruitenberg A, Hofman A, Stricker BH, Breteler MM (2001). Antihypertensive drugs and incidence of dementia: the Rotterdam Study. Neurobiol Aging.

[20] Paganini-Hill A (2012). Hypertension and dementia in the elderly: the leisure world cohort study. Int J Hypertens.

[21] Rozzini L, Vicini Chilovi B, Bellelli G, Bertoletti E, Trabucchi M, Padovani A (2005). Effects of cholinesterase inhibitors appear greater in patients on established antihypertensive therapy. Int J Geriatr Psychiatry.

[22] Pellfolk T, Gustafsson T, Gustafson Y, Karlsson S (2009). Risk factors for falls among residents with dementia living in group dwellings. Int Psychogeriatr.

[23] Whitney J, Close JC, Jackson SH, Lord SR (2012). Understanding risk of falls in people with cognitive impairment living in residential care. J Am Med Dir Assoc.

[24] Moonen JE, Foster-Dingley JC, de Ruijter W, van der Grond J, Bertens AS, van Buchem MA (2015). Effect of discontinuation of antihypertensive treatment in elderly people on cognitive functioning—the DANTE Study Leiden: a randomized clinical trial. JAMA Intern Med.

[25] Cooper C, Ketley D, Livingston G (2014). Systematic review and meta-analysis to estimate potential recruitment to dementia intervention studies. Int J Geriatr Psychiatry.

[26] Reeve E, Bell JS, Hilmer SN (2015). Barriers to optimising prescribing and deprescribing in older adults with dementia: a narrative review. Curr Clin Pharmacol.

[27] Harrison JK, Van Der Wardt V, Conroy SP, Stott DJ, Dening T, Gordon AL (2016). New horizons: the management of hypertension in people with dementia. Age Ageing.

